# Novel Real-Time Temperature Diagnosis of Conventional Hot-Embossing Process Using an Ultrasonic Transducer

**DOI:** 10.3390/s141019493

**Published:** 2014-10-17

**Authors:** Chin-Chi Cheng, Sen-Yeu Yang, Dasheng Lee

**Affiliations:** 1 Department of Energy and Refrigerating Air-Conditioning Engineering, National Taipei University of Technology, Taipei, Taiwan; E-Mail: f11167@ntut.edu.tw; 2 Department of Mechanical Engineering, National Taiwan University, Taipei, Taiwan; E-Mail: syyang@ntu.edu.tw

**Keywords:** high temperature ultrasonic transducer (HTUT), conventional hot embossing, ultrasonic velocity, replication, process diagnosis

## Abstract

This paper presents an integrated high temperature ultrasonic transducer (HTUT) on a sensor insert and its application for real-time diagnostics of the conventional hot embossing process to fabricate V-cut patterns. The sensor was directly deposited onto the sensor insert of the hot embossing mold by using a sol-gel spray technique. It could operate at temperatures higher than 400 °C and uses an ultrasonic pulse-echo technique. The ultrasonic velocity could indicate the three statuses of the hot embossing process and also evaluate the replication of V-cut patterns on a plastic plate under various processing conditions. The progression of the process, including mold closure, plastic plate softening, cooling and plate detachment inside the mold, was clearly observed using ultrasound. For an ultrasonic velocity range from 2197.4 to 2435.9 m/s, the height of the V-cut pattern decreased from 23.0 to 3.2 μm linearly, with a ratio of −0.078 μm/(m/s). The incompleteness of the replication of the V-cut patterns could be indirectly observed by the ultrasonic signals. This study demonstrates the effectiveness of the ultrasonic sensors and technology for diagnosing the replicating condition of microstructures during the conventional hot embossing process.

## 1. Introduction

**Figure 1.**
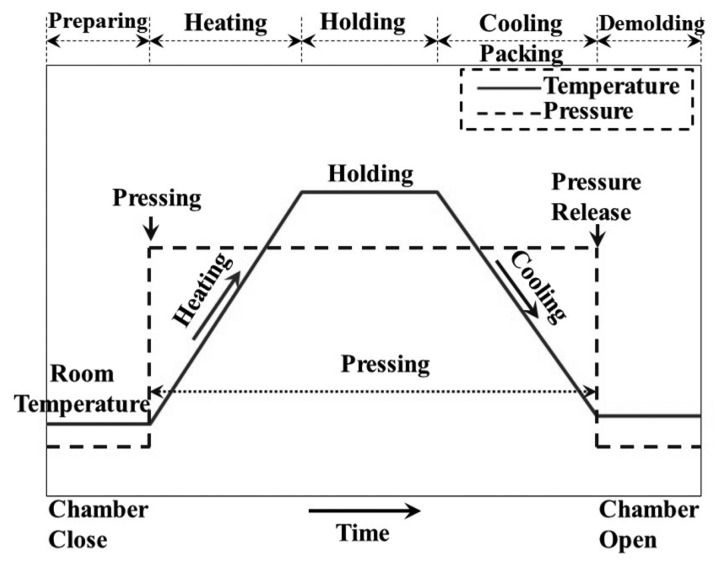
Procedures of the conventional hot embossing process, with the pressure (**Solid Line**) and temperature profiles (**Dashed Line**) illustrated; the procedures, including preparation, heating, holding, cooling and demolding stages.

Hot embossing is one of the most widely used techniques and productive methods for the parallel replication of precise micro-or nano-features on a polymeric substrate at low cost [1]. The identical structures, such as arrays of hole, dot, wire and ring transistors, can be reproduced on substrates with a single master or stamp [2]. This technique is widely applied for transferring micro-features to thermoplastic films for optical, microfluidic and bio-chip applications, because of its low cost for the molds, high replication accuracy for the micro-features and simple operation in the tool and process setup [3]. The hot embossing process is illustrated in Figure 1, with the pressure profile denoted with the solid line and the temperature profile with the dashed line. The first step is the preparation. The mold with a microstructure is placed above the plastic plate substrate to form a stack, and the stack is then placed on the lower heating/cooling plate. During the hot embossing operation, the lower heating/cooling plate is first moved up, so that the mold/substrate stack is heated and pressed by the upper and lower heating plates. After a sufficient holding period for the patterns in the mold to be fully transferred onto the substrate, the stack is cooled down to below its glass transition temperature with circulating water in the plates. For pressing, the mold/substrate stack is compressed by a specific pressure. The pressure can be provided by a pneumatic cylinder [4], hydraulic cylinder [5] or linear screw/motor devices [6]. The final step is demolding. After the stack is cool, the lower heating/cooling plate with the stack is moved down and the pressure is released. The mold can be separated from the plate. A plastic plate with the replicated microstructures can be obtained.

Hardt *et al.* [7] found that mold temperature, forming force, forming rate, hold time, *etc.*, were the parameters affecting the replication of microstructures in the hot embossed substrate. Roos *et al.* [8] confirmed that more uniform replication and less defects could be reached with a vacuum. They also identified that the temperature and hold time were the most critical parameters. The pressure was not as critical, as long as it was above a certain threshold. Therefore, it was important to monitor the temperature during hot embossing. Currently, the widely used process monitoring and diagnostic tools deploy temperature sensors for online measurements. However, in most cases, this may not be practical for the hot embossing process. If the sensors were mounted and in direct contact with the substrate, they may leave detrimental marks on the parts. The ultrasonic technique is a nondestructive and non-intrusive method for the real-time diagnosis of the polymer processes [9]. The basic signatures of ultrasonic signals, such as velocity, attenuation, reflection and transmission coefficients, scatter signals from materials, have unique relationships with the process dynamics [10], material characteristics [11] and product quality [12]. Ultrasonic signals can reveal the temperature of the material [13–15]. However, to the authors' knowledge, this has not been utilized for the diagnosis of the conventional hot embossing process. This study attempts to apply ultrasonic techniques for the real-time diagnosis of the conventional hot embossing process. In this study, a high temperature ultrasonic transducer (HTUT) is to be integrated onto the sensor insert of a hot embossing mold. During the hot embossing operation for replicating a V-cut pattern from the mold to substrates at various temperatures, the process is to be monitored and diagnosed by ultrasonic technology.

## 2. Development of Ultrasonic-Assisted Diagnosis System

**Figure 2.**
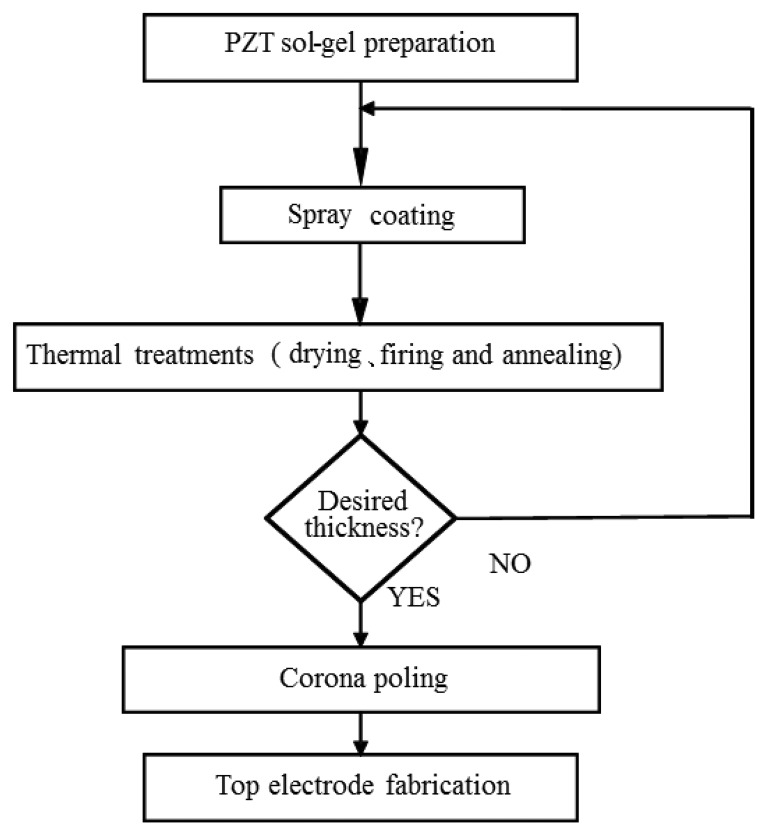
Flow chart of lead zirconate titanate (PZT) film fabrication procedures by the sol-gel spray coating technique, including sol-gel preparation, spray coating, thermal treatment, corona poling and fabrication of the top electrode.

Since the sensors have to sustain a temperature as high as 225 °C for most polymer processes, HTUTs are developed to realize the ultrasonic diagnosis. The procedure for fabricating the HTUT by a sol-gel spray technique is described in Figure 2 [16–18]. The piezoelectric bismuth titanate (BIT) powders were dispersed into lead zirconate titanate (PZT) solution to achieve the gel. BIT was chosen because of its high Curie temperature, 675 °C, and reasonable piezoelectric strength. PZT was selected due to its high dielectric constant, so that during poling and ultrasonic application, the electrical signal can be mainly applied across the film. A handheld air gun was then used to spray the sol-gel composite directly onto the insert. After spray coating, the thermal treatments of drying, firing and annealing with the optimal time duration were practiced. Multiple layers were made in order to reach the desired thickness. The 40–200 μm-thick films could provide the desired center frequency in the range of 2–30 MHz. This frequency range is commonly preferred for NDE of metals and industrial material process monitoring, because of its sufficient ranging resolution and acceptable ultrasonic attenuation in metals and polymers. The films were then electrically poled using the corona discharging technique to achieve piezoelectricity. Afterwards, a silver paste was used to form the top electrode. The silver paste has been tested, and its operating temperature was above 440 °C. In this experiment, the thickness of the PZT film was 90 μm and the top electrode diameter was 5 mm. The HTUT was applicable at temperatures higher than 400 °C without an ultrasonic couplant. It could be operated in a medium megahertz (MHz) frequency range with a sufficient frequency band width and had a sufficient piezoelectric strength and signal-to-noise (SNR) ratio.

**Figure 3.**
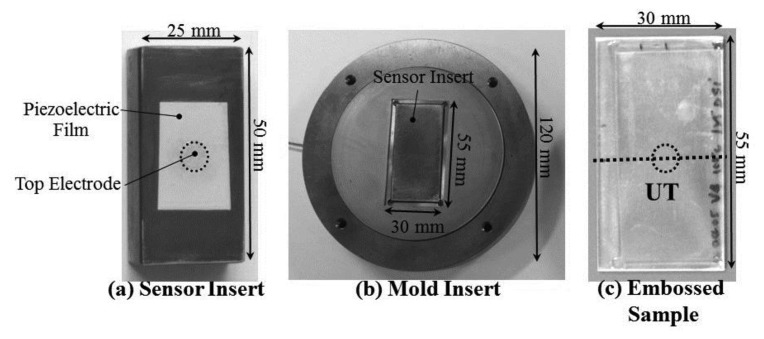
Photographs of (**a**) the sensor insert with HTUT; (**b**) the mold insert and (**c**) the embossed sample with the scanning path denoted by the dotted line passing the UT location.

According to the mold size of the hot embossing machine, the dimension of the sensor insert was designed as a rectangular shape: 25 mm in width, 50 mm in length and 12 mm in height, as shown in Figure 3a. By replacing the sensor insert, the shape and dimensions of the molded part can be easily modified to meet the customer's demands. The sensor insert was installed into the mold insert with a circle shape, as shown in Figure 3b. The probing end of the HTUT sensor insert was flush with the mold cavity surface. The dimensions of the mold cavity were 30 mm in width and 55 mm in length for a rectangular plate. The HTUT was vertically aligned on the center line of the cavity. Figure 3c shows the photo of the embossed sample with the scanning path denoted by the dotted line passing the UT location. The dimensions of the embossed plate were 30 mm wide, 55 mm long and 2 mm thick.

## 3. Experimental Setup

**Figure 4.**
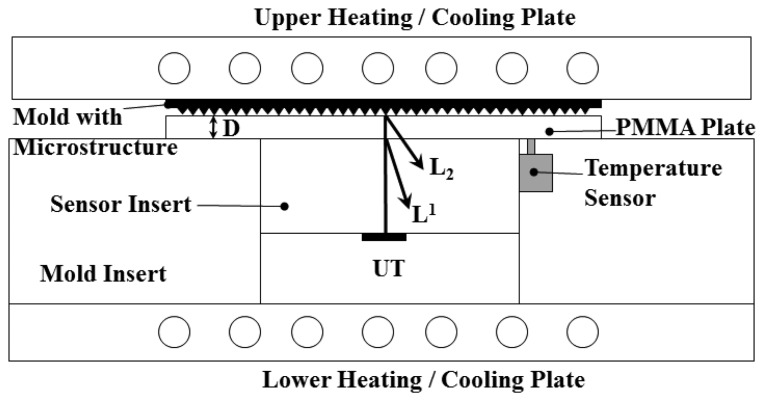
Schematic drawing of the cross-section of the experimental setup: The heating/cooling plates, the HTUT sensor insert with the mold and the mold insert, indicating the ultrasonic transmitting path.

In this experiment, a 35-ton hot-embossing machine (Shan Yung, Taipei, Taiwan), equipped with upper and lower heating/cooling plates, was used. The hot plate was heated by the heater strip inside the plate and was cooled by circulating water. The lower heating/cooling plate could be moved up and down by the hydraulic cylinder. The HTUT sensor insert with the mold insert was installed on the lower heating/cooling plate of the hot-embossing machine. A schematic view of the experimental setup with the sensor insert, mold insert and heating plate is displayed in Figure 4. As shown in Figure 4, when electric pulses were applied to the piezoelectric film through the top and bottom electrodes, where the insert itself served as the bottom electrode, ultrasonic waves were excited and transmitted into the insert. L^1^ denotes the 1st round trip longitudinal-wave ultrasonic echo reflected from the internal surface of the mold cavity, and L_2_ is the 1st echo propagating in the plastic plate and reflected from the plastic plate/mold interface. It is noted that the L_2_ echo appears only when the plastic plate is soft and in contact with the HTUT insert. The L^1^ and L_2_ echoes will be used to monitor the embossing cycle and polymer state. A temperature sensor (K-type thermocouple, Omega, Stamford, CT, US) was mounted beside the HTUT (abbreviated as UT) insert and made flush with the top surface of the mold insert. The temperature would be measured for a comparison with the ultrasonic signals during the embossing process.

**Figure 5.**
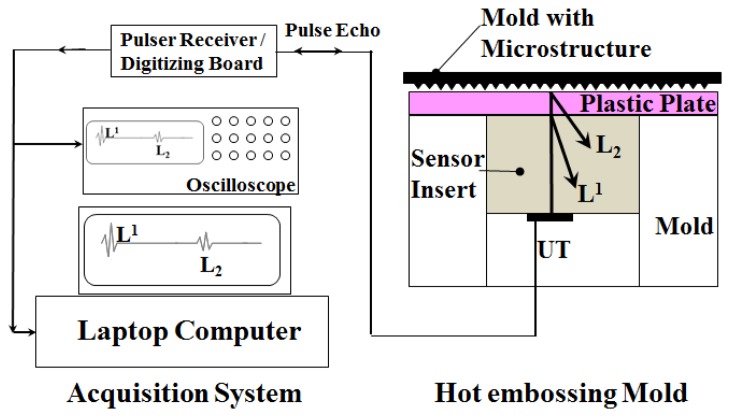
Schematic view of the sensor insert and mold in the hot embossing machine with UT and the data acquisition system for the ultrasonic diagnosis of the hot embossing process using the ultrasonic pulse-echo technique.

The electrical connection between the sensor insert and ultrasonic data acquisition system for the diagnosis of the hot embossing process is shown in Figure 5. The ultrasonic data acquisition system (Pace Simulation Inc., Montreal, QC, Canada) is composed of a one-channel signal pulsing-receiving system, a 12-bit single-channel digitizing board and a laptop with a data acquisition and analysis program by LabVIEW for acquiring, demonstrating and storing the data. The pulsing-receiving system (Tecscan system Inc., Boucherville, QC, Canada) is a broadband, negative spike pulser and broadband receiver. It can be applied in reflection or transmission mode. For the pulser, the driving voltage is 50∼300 V and the pulse duration is 30∼500 ns. For the receiver, the broad bandwidth is 300 kHz∼60 MHz. The digitizing board (National Instrument, Austin, TX, USA) has a maximum sampling rate of 50 MHz for dual channels. It has an 8-MB on-board memory and 8-bit A/D resolution. An optional oscilloscope (Angilent, DSOX3012A, Santa Clara, CA, USA) is also necessary for monitoring the signals in real time. The data acquisition and analysis program by LabVIEW combines the computation, display and connective capabilities of computers to provide robust and flexible instrumentation functions. The acquisition configurations, including operation mode, triggering, mapping, converting and data recording requests, can be easily set up for recording the ultrasonic signatures and calculating the sound speed. All of the experiments presented in this study were conducted in the ultrasonic pulse-echo mode. The acquisition rate was 2 Hz in this paper.

**Figure 6.**
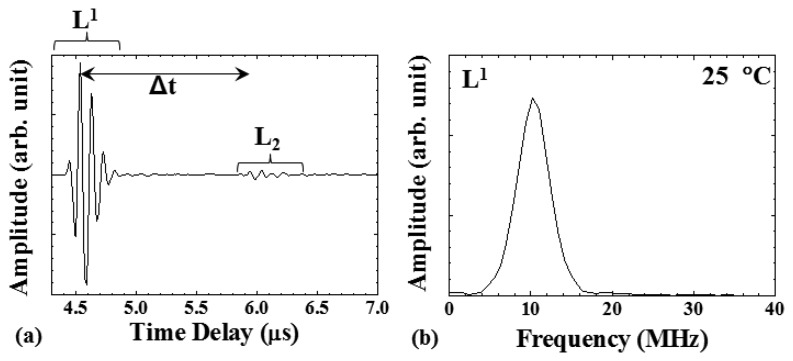
(**a**) Typical ultrasonic signals acquired by UT; (**b**) the frequency spectrum of L^1^ signal. The center frequency of the L^1^ echo was 10.2 MHz, and the 3-dB bandwidth was 4.6 MHz.

Figure 6a shows the typical signals acquired with the UT (in Figure 3a during the embossing process), when the plastic plate is soft and in contact with the HTUT insert. As can be seen, the L^1^ echo, reflected at the insert end/polymer plate or air interface, appeared at 4.5 μs and remained during the entire cycle. When the plastic plate was soft and in contact with the UT insert, the L_2_ echo, propagating in the plastic plate and reflected at the plastic plate/mold interface, was observed at 6.0 μs. The time delay difference between the L^1^ and L_2_ echoes was denoted as Δt. Figure 6b shows the frequency spectrum of the L^1^ echo in Figure 6a. The center frequency of the L^1^ echo was 10.2 MHz, and the 3-dB bandwidth was 4.6 MHz. The signal-to-noise ratio (SNR) for the first round trip echo, L^1^, was 39 dB.

**Figure 7.**
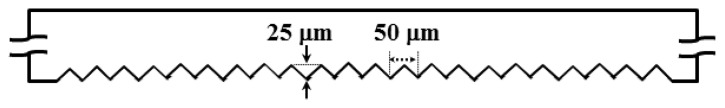
Schematic drawing of the mold with V-cut patterns. The sizes of the V-cut pattern were 25 μm high and 50 μm wide.

The machine settings in the experiments were as follows: The heating temperatures were 80 °C, 100 °C and 120 °C; the hydraulic pressure in the pressurizing stage was 1 MPa; the holding period was 5 min. The utilized substrate was a polymethylmethacrylate (PMMA) plate. The V-cut patterns on the mold surface, as shown in Figure 7, would be replicated on the surface of the plastic plate during the embossing process. The dimensions of the mold were 30 mm wide, 60 mm long and 0.5 mm thick. The sizes of the V-cut pattern were 25 μm high and 50 μm wide. The replicated V-cut pattern on the surface of the plastic plate was scanned by the laser scanning confocal microscope, in order to be compared with that measured by the ultrasonic technique in the following section.

## 4. Results and Discussions

### 4.1. Embossing Process Diagnosed by Ultrasonic Signatures

**Figure 8.**
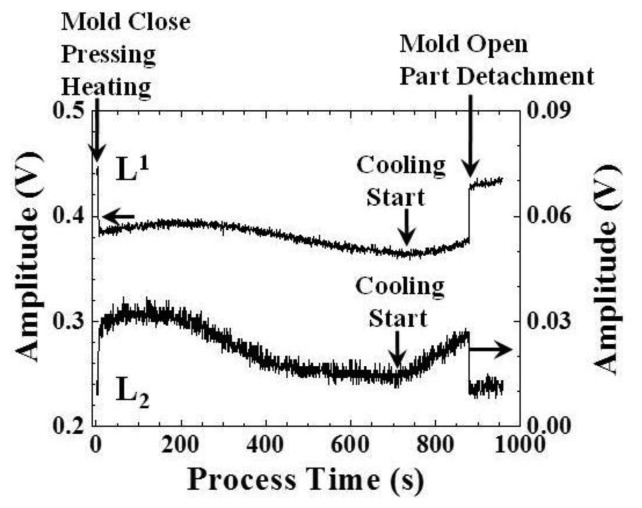
Amplitude variations of the ultrasonic L^1^ and L_2_ echoes under a temperature setting of 100 °C, indicating the mold closure, pressing, heating, cooling and mold opening during the embossing process.

In order to investigate the correlation between the ultrasonic signals observed and the embossing cycle, the amplitude values of the L^1^ and L_2_ echoes in Figure 6a with respect to the process time were obtained. The results are presented in Figure 8. At a process time of 7s, the mold closed and was pressed by the hot plates with a pressure of 1 MPa, and then, the plastic plate started to heat till a desired temperature of 100 °C. At this moment, the amplitude of the L^1^ echo decreased and the amplitude of the L_2_ echo increased, due to the fact that a part of the ultrasonic energy was transmitted into the plastic plate through the sensor insert/plastic plate interface. At a process time of 230 s, the amplitude of the L^1^ echo decreased again, due to the softening of the plastic plate.

At process times of 733 and 716 s, the amplitudes of the L^1^ and L_2_ echoes started to increase, respectively, because of the cooling of the plate. There was a time difference between the ultrasonic L^1^ and L_2_ echoes, indicating that the ultrasonic L_2_ echo responded earlier than the L^1^ echo. This may be due to the temperature gradient in the mold insert and plastic plate. At a process time of 880 s, the amplitude of the L^1^ echo recovered almost to the original value, and the amplitude of the L_2_ echo decreased to the noise level. This was because the mold was opened and the part was detached from the mold and sensor insert. The transducer sensitivity may be affected by the temperature variation [19]. However, the changes of the transducer sensitivity and ultrasonic echo amplitude may benefit for indicating the status variation clearly, especially the cooling start.

**Figure 9.**
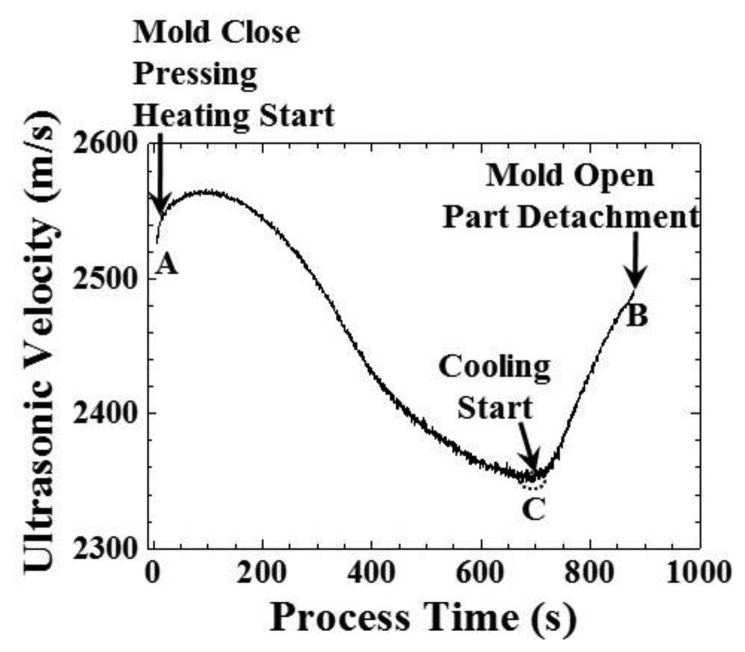
Variation of the ultrasonic velocity under the temperature setting of 100 °C, indicating the mold closure, pressing, heating, cooling and mold opening during the embossing process.

During the embossing process, the solidification would affect the replicating effect of the microstructure from the mold to the plastic plate. Ultrasonic velocity may be one of the candidates to indicate the polymer state in the mold, because of its close relationship with the temperature. The ultrasonic velocity in the polymer plate could be calculated according to the following equation:

(1) $${\text{v}}_{\text{m}}=2\text{D}/\Delta \text{t}$$

where D is the thickness of the plastic plate in Figure 4 and Δt is the time delay between the ultrasonic L^1^ and L_2_ echoes in Figure 6a. The result is shown in Figure 9.

In Figure 9, the ultrasonic velocity appeared at a process time of 7 s, when mold was closed, pressed and heated, denoted as the A status. From a process time of 7 to 93 s, the ultrasonic velocity increased from 2525.8 to 2564.8 m/s, indicating that the temperature decreased due to the heat absorbed by the plastic plate. From a process time of 93 to 716 s, the ultrasonic velocity decreased from 2564.8 to 2352.9 m/s, indicating that the temperature increased during the heating and holding stages. From a process time of 716 to 880 s, the ultrasonic velocity increased from 2352.9 to 2489.6 m/s, indicating that the temperature decreased during the cooling stage. At a process time of 716 s, when the system started to cool down, denoted as the C status, the ultrasonic velocity in the polymer could be utilized for indicating the replication of the V-cut microstructure in the following section. At a process time of 880 s, the mold opened, the molded part detached and the ultrasonic velocity disappeared, due to the vanishing of the amplitude of the L_2_ echo, denoted as the B status. The three statuses of the conventional hot embossing process could be diagnosed through the amplitude and velocity variation of the ultrasonic signals. The advantage of the ultrasound amplitude over the velocity is that it indicates the three statuses of the conventional hot embossing process directly (it is not necessary for further calculation).

### 4.2. Embossing Process Diagnosed by Temperature

**Figure 10.**
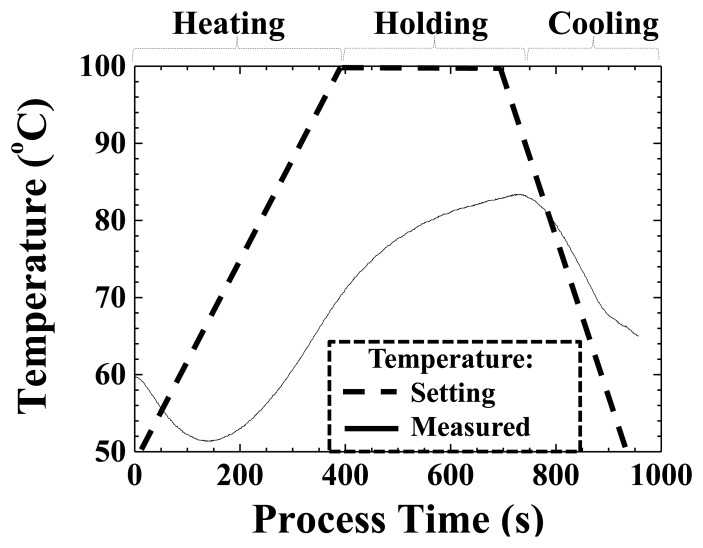
Variations of the setting temperature (**Dotted Line**) of the heating coil and the measured surface temperature (**Solid Line**) of the plastic plate with the temperature sensor for comparison. The setting temperature was 100 °C.

Temperature variation during the process has a close relationship with the state of the plastic plate, and it would affect the replication of the pattern from the mold to the surface of the plastic plate. The surface temperature of the plastic plate as recorded by a temperature sensor installed beside the HTUT insert is shown in Figure 10. In Figure 10, the dashed line is the setting temperature of the heating coil and the solid line is the measured surface temperature of the plastic plate. The hot plate was heated from a process time of 0 to 389 s and kept constant at 100 °C throughout the holding period. From 688 s, the plate was cooled down by circulating water to room temperature. The surface temperature of the plastic plate decreased from 60 to 51.4 °C in the beginning, due to the absorption of heat by the plastic plate. From 136 s to 737 s, the surface temperature of the plastic plate increased monotonically from 51.4 to 83.4 °C. After 737 s, it started to decrease until a cycle time of 957 s. One may notice that the pattern of the temperature in Figure 10 is similar to the inverse of the ultrasonic velocity in Figure 9. This phenomenon may be explained according to Morath and Maris's research results [20]. They presented that the ultrasonic velocity and polymer (PMMA) temperature had the following relationship in their experimental conditions:

(2) $$\frac{1}{{\text{v}}_{\text{m}}}\frac{{\text{dv}}_{\text{m}}}{\text{dT}}=-5*10^{-4}$$

where v_m_ was the ultrasonic velocity in the polymer (PMMA), T was the temperature (K) and the applied temperature range was from 80 to 300 K. The similar phenomenon could also be observed from the research results of Ono *et al.* [21]. The ultrasonic velocity had a negative piecewise linear correlation with a polymer (PMMA) temperature from 55 to 250 °C in their experimental conditions.

### 4.3. Height of Microstructures Evaluated by Ultrasonic Velocity

**Figure 11.**
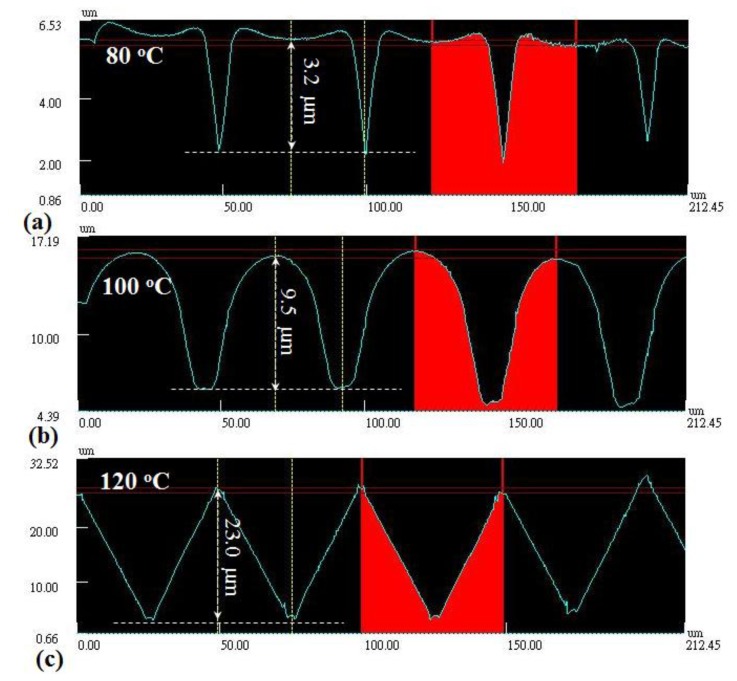
Cross-section views of the embossed samples under temperature settings of (**a**) 80 °C; (**b**) 100 °C and (**c**) 120 °C, scanned by the laser scanning confocal microscope. The height differences of the marked patterns were 3.2, 9.5 and 23.0 μm for the temperature settings of 80, 100 and 120 °C, respectively.

The V-cut patterns of the mold would be replicated to the surface of the plastic plate during the embossing process. The replicated pattern on the surface of the plastic plate was scanned by the laser scanning confocal microscope. The cross-section views of the embossed samples fabricated at 80, 100 and 120 °C are shown in Figure 11a–c, respectively. The scanning path on the sample surface was located on the UT location, as shown in Figure 3c. The height differences of the marked patterns were 3.2, 9.5 and 23.0 μm for temperature settings of 80, 100 and 120 °C, respectively. The height difference of the scanned pattern represented the replication of the microstructure. It seemed that the higher temperature setting would cause a larger height difference and a better replication of the microstructure.

**Figure 12.**
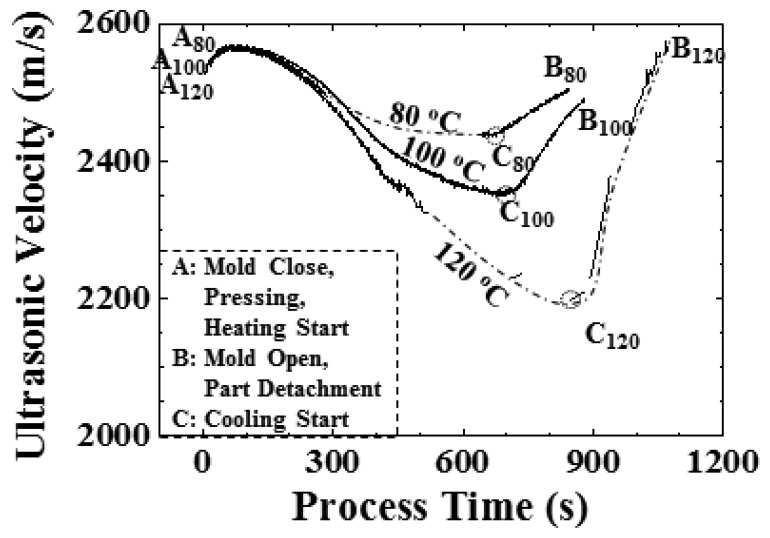
Variation of ultrasonic velocities under temperature settings of 80, 100 and 120 °C, indicating the mold closure, pressing, heating, cooling and mold opening during the hot embossing process. Higher temperature settings caused a lower ultrasonic velocity at the cooling starting point, as well as a longer processing period.

In order to further understand the results presented in Figure 11, the solidification of the hot embossing process will be diagnosed under various temperature settings, due to the solidification affecting the replication of the microstructure significantly during the embossing process. The ultrasonic velocity would be utilized to indicate the polymer state in the mold, because of its close relationship with the temperature. The ultrasonic velocities under various temperature settings (80, 100 and 120 °C) during the hot embossing process are shown in Figure 12. In Figure 12, the patterns of the ultrasonic velocities are similar to those in Figure 9, and the three processing statuses are denoted as follows: A status: Mold closure, pressing and heating start; B status: Mold opening and part detachment; C status: Cooling start. The subscripts of 80, 100 and 120 are the setting temperatures. Certain periods of the ultrasonic velocity under the temperature settings of 80 and 120 °C were missed in Figure 12, due to the absence of the L_2_ echo in the high attenuation region. These periods are connected by the dotted lines. For the three processing statuses, the related ultrasonic velocity and the displayed time under the temperature settings of 80, 100 and 120 °C are illustrated in Table 1. In Figure 12 and Table 1, for the A status, the ultrasound appeared within the process time range of 10 s, and the ultrasonic velocity difference was less than 15.3 m/s, indicating the similar initial mold temperature. For the B status, the ultrasound under a temperature setting of 120 °C disappeared later, and the ultrasonic velocity was higher than other two temperature settings, indicating the longer processing period and lower mold opening temperature. For the C status, the displayed time increased with the setting temperature, indicating that the higher temperature setting would take a longer period of time to reach the steady state. Comparing the ultrasonic velocity at the C status in Figure 12 with the height difference of the microstructure in Figure 11 under the different temperature settings, there existed some relationship that would be useful for indicating the replication of the microstructure during the embossing process.

**Figure 13.**
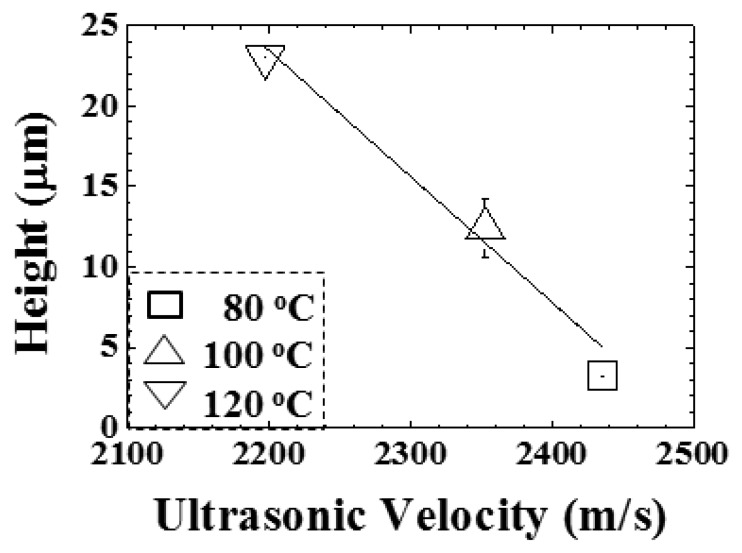
Height differences of the V-cut pattern under various temperature settings indicated by the ultrasonic velocity at the C status (cooling-start), with a ratio of −0.078 μm/(m/s). The ultrasonic velocity may be utilized to indicate the replication of the V-cut pattern under the various temperature settings.

In order to estimate the replication of the microstructure by the ultrasonic velocity, the ultrasonic velocity at the C status in Figure 12 is compared with the height difference of the V-cut patterns in Figure 11. The height difference of the V-cut patterns with respect to ultrasonic velocity is shown in Figure 13. The temperature settings of 80, 100 and 120 °C are represented by symbols of □, Δ and ∇, respectively. In Figure 13, in the ultrasonic velocity range from 2197.4 to 2435.9 m/s, the height difference of the V-cut pattern decreased from 23.0 to 3.2 μm linearly, with a ratio of −0.078 μm/(m/s). The height difference of the V-cut pattern could be expressed as:

(3) $$\Delta \text{h}=195.37-0.078*{\text{v}}_{\text{m}}$$

where Δh is the height difference of the V-cut pattern and v_m_ is the ultrasonic velocity in the polymer at the C status. The linearity of Equation (3) could be held within the temperature range from 80 to 120 °C or the ultrasonic velocity range from 2200 to 2450 m/s, under our experimental settings. The higher temperature setting would cause larger height differences in the microstructure, as shown in Figure 11, and lower ultrasonic velocity, as shown in Figure 12. These results demonstrate that the ultrasonic velocity at the C status may be one of the options to indicate the replication of the V-cut pattern under the various temperature settings.

## 5. Conclusions

The diagnosis of the hot embossing process is essential to enhance the precise replication of microstructures. The ultrasonic transducer technology is one of the most effective and efficient tools for real-time, non-intrusive and non-destructive monitoring. In this study, one integrated HTUT was utilized on a sensor insert for the diagnosis of the conventional hot embossing process. The progression of the process, including mold closure, plastic plate softening, cooling and plate detachment inside the mold, was clearly observed using ultrasound. Ultrasonic velocity could not only indicate the three statuses of the process, but could also evaluate the replication of the V-cut patterns on the plastic plate under various temperature settings. For the ultrasonic velocity range from 2197.4 to 2435.9 m/s, the height differences of the V-cut patterns decreased from 23.0 to 3.2 μm linearly, with a ratio of −0.078 μm/(m/s). The replication incompleteness of the V-cut patterns could be indirectly observed by the ultrasonic signals. This study demonstrates the potential of ultrasonic sensors and technology for diagnosing the replicating condition of microstructures during the conventional hot embossing process.

## Figures and Tables

**Table 1.** Ultrasonic velocities and times for the three processing statuses under the temperature settings of 80, 100 and 120 °C.

**Status Temperature (°C)**	**A**		**B**		**C**	

	**Time (s)**	**Velocity (m/s)**	**Time (s)**	**Velocity (m/s)**	**Time (s)**	**Velocity (m/s)**
80	16	2539.1	845	2505.6	667	2435.9
100	7	2525.8	880	2489.6	716	2352.9
120	17	2541.1	1066	2574.2	847	2197.4
